# Targeting polyIC to EGFR over-expressing cells using a dsRNA binding protein domain tethered to EGF

**DOI:** 10.1371/journal.pone.0162321

**Published:** 2016-09-06

**Authors:** Nufar Edinger, Mario Lebendiker, Shoshana Klein, Maya Zigler, Yael Langut, Alexander Levitzki

**Affiliations:** 1 Unit of Cellular Signaling, Department of Biological Chemistry, The Alexander Silberman Institute of Life Sciences, The Hebrew University of Jerusalem, Jerusalem, Israel; 2 Protein Purification Unit, Wolfson Center for Applied Structural Biology, The Alexander Silberman Institute of Life Sciences, The Hebrew University of Jerusalem, Jerusalem, Israel; Consiglio Nazionale delle Ricerche, ITALY

## Abstract

Selective delivery of drugs to tumor cells can increase potency and reduce toxicity. In this study, we describe a novel recombinant chimeric protein, dsRBEC, which can bind polyIC and deliver it selectively into EGFR over-expressing tumor cells. dsRBEC, comprises the dsRNA binding domain (dsRBD) of human PKR (hPKR), which serves as the polyIC binding moiety, fused to human EGF (hEGF), the targeting moiety. dsRBEC shows high affinity towards EGFR and triggers ligand-induced endocytosis of the receptor, thus leading to the selective internalization of polyIC into EGFR over-expressing tumor cells. The targeted delivery of polyIC by dsRBEC induced cellular apoptosis and the secretion of IFN-β and other pro-inflammatory cytokines. dsRBEC-delivered polyIC is much more potent than naked polyIC and is expected to reduce the toxicity caused by systemic delivery of polyIC.

## Introduction

Selective delivery of drugs to tumor cells can improve efficacy and reduce toxicity. Selectivity can be obtained by utilizing a drug vehicle that can distinguish between the targeted malignant cells and untargeted non-malignant cells. High specificity towards cancer can be programmed into recombinant proteins by fusing targeting moieties and drug binding moieties. The targeting moiety must recognize cell surface molecules that are uniquely expressed on cancer cells but not on non-cancerous cells or are over-expressed in cancer cells as compared to their normal counterparts. One appropriate target is the Epidermal Growth Factor Receptor (EGFR), which is over-expressed in multiple types of human cancer and is usually associated with aggressive disease and low survival rate [1]. EGFR over-expression can be utilized to selectively deliver high quantities of polyinosine/polycytosine (polyIC) into tumor cells, while leaving normal cells unaffected, due to the low amounts of polyIC delivered. PolyIC is an attractive anti-tumor agent, as it can induce cancer cell apoptosis by activating Toll Like Receptor 3 (TLR3) in cancer cells [2–6]. Furthermore, TLR3 activation by polyIC triggers the induction of cytokines, chemokines and other pro-inflammatory mediators [7–10], thus reinstating anti-tumor immunity [11,12]. However, the use of polyIC is limited by its extreme toxicity and inefficient cellular uptake when delivered systemically [13,14].

In order to limit toxicity and increase cellular uptake we have been developing vehicles for the targeted delivery of polyIC directly to tumors. In our previous studies we employed chemical vectors that bind PolyIC electrostatically, and utilize EGF or anti-HER2 affibody as homing entities towards EGFR or HER2 [15–18].

**Fig 1:**
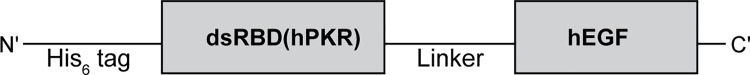
Schematic description of the chimeric protein, dsRBEC. The dsRBD of hPKR enables polyIC binding and is fused via a linker to the homing moiety, hEGF. The His_6_ tag facilitates purification on Ni Sepharose.

In this report we describe an alternative approach, namely, the generation of a chimeric protein molecule that can deliver polyIC to EGFR over-expressing cells. The chimeric protein, dsRBEC (*dsRB*D-*E*GF-*C*himera) is composed of the dsRNA-binding domain (dsRBD) of hPKR (residues 1–197) fused via a linker to hEGF (Fig 1). The dsRBD of hPKR is composed of two copies of a dsRNA binding motif (dsRBM), which are connected through a flexible linker and can interact with dsRNA in a non-sequence specific manner [19,20]. This dsRBD is the polyIC binding moiety of dsRBEC. EGF is used as a targeting moiety, which selectively binds EGFR and induces endocytosis. As compared with the polymeric chemical vector, the chimeric protein is precisely defined and can be produced simply and inexpensively. Unlike current anti-EGFR therapies, which target the activity of the receptor, our therapy does not inhibit the EGFR pathway but exploits the over-expression of the receptor for selectivity and for cellular entry.

In this paper we describe the purification of the active chimera and its activity in tumor cells.

## Materials and Methods

### Cell culture

A431 cells were grown in DMEM supplemented with 10% fetal calf serum (FCS), penicillin and streptomycin. MDA-MB-468 and MCF7 cells were grown in RPMI 1640 medium supplemented with 10% FCS, penicillin and streptomycin.

### Cloning

The chimeric gene encoding dsRBEC was constructed as follows: The dsRBD of hPKR (nucleotides 558–1057, **NM_002759.3**) was fused to the nucleic acid sequence of hEGF (GenBank: **M11936.1**). The 3’ sequence of the dsRBD (nucleotides 1028–1057) was changed to GGCCAAACTGGCCTATCTGCAGATCTTATC to optimize codon usage and to introduce new restriction sites, and a linker (GGCGTGTTCGGGATCCGCC GGCAACCGTGTCCGTCGGAGCGTGGGCAGCTCGAATGGA), encoding ACSGSA CSGSAGNRVRRSVGSSNG, was introduced before the hEGF moiety. The chimera was cloned into the bacterial expression vector pET28a (Novagen), between restriction sites NdeI and HindIII, thus inserting a Hexa His tag at the N-terminus of the chimera.

### dsRBEC expression

*E*.*coli* BL21(DE3)/CodonPlus RIL (Stratagene) carrying the pET28a-His_6_-dsRBEC plasmid was grown in 2xYT [21] supplemented with 1% glucose, 25μg/ml chloramphenicol, and 30μg/ml kanamycin at 37°C to OD600~0.6. At this point, the bacteria were moved to 23°C. Protein expression was induced by adding 0.5mM Isopropyl-β-D-thiogalactopyranoside (IPTG), and the culture was incubated at 23°C for 6 hours longer. The bacterial culture was then centrifuged at 5000xg for 10 minutes and the pellet was stored at -80°C until further applications.

### Small scale purification and RNA contamination analysis

The pellet from 10 ml bacterial culture was resuspended in 1 ml lysis buffer (20mM Hepes pH 7.5, 0.5M NaCl, 10% glycerol, 10mM imidazole) and disrupted using a LV1 microfluidizer (Microfluidics). Following 15 minutes centrifugation at 15,000×g and 4°C, the cleared supernatant was loaded onto 50 μl equilibrated Ni Sepharose High Performance beads (GE Healthcare Life Sciences) and rotated for 1 hour at 4°C. Following two washes with lysis buffer, the bound protein was eluted with 200 μL elution buffer (20mM Hepes pH 7.5, 0.5M NaCl, 10% glycerol, 500mM imidazole). Samples from each step (total lysate, soluble fraction, unbound fraction and eluate) were subjected to SDS–PAGE (15% polyacrylamide). The gel was stained with InstantBlue Coomassie based gel stain (Expedeon) or transferred to nitrocellulose membranes for western analysis using anti-His tag antibody (LifeTein, # LT0426, 1:1000 dilution). To visualize nucleic acid contamination of the protein, 30μl of the eluted protein were electrophoresed on a 1% agarose gel. Where relevant, the protein was treated with RNase A (10μg/ml) for 30 minutes at 37°C prior to agarose gel electrophoresis. The gel was stained with ethidium bromide following electrophoresis. For purification under denaturing conditions, the bacterial pellet was resuspended with lysis buffer containing 4M urea, and was incubated at 4°C for 1.5 hours prior to centrifugation.

### On-column purification and renaturation

The pellet from 500 ml of bacterial culture was resuspended with 40 ml lysis buffer supplemented with 4M urea and disrupted using a LV1 microfluidizer. The lysate was incubated at 4°C for 1.5 hours, and cleared by centrifugation for 30 minutes at 15,000×g at 4°C. The clear supernatant was loaded onto 4 ml equilibrated Ni Sepharose beads and incubated for an additional hour at 4°C in a 50 ml tube. The beads were then loaded onto a 4 ml C 10/10 column (GE Healthcare) and connected to an AKTA Explorer system (GE Healthcare). The protein was refolded by gradually reducing the concentration of urea. A gradient program was used with Buffer A (20mM Hepes pH 7.5, 0.5M NaCl, 10mM imidazole, 10% glycerol, 4M urea) and Buffer B (20mM Hepes pH 7.5, 0.5M NaCl, 10mM imidazole, 10% glycerol). The gradient was programmed to reach 100% B in 30 column volumes (CV) at 0.2 ml/minute flow. The column was washed with 4 CV of buffer (20mM Hepes pH 7.5, 0.5M NaCl, 10% glycerol) containing 25mM imidazole and with another 4 CV of buffer with 50mM imidazole. The chimera was eluted in the same buffer, to which imidazole had been added to 500mM. The protein eluted from the Ni Sepharose column was loaded onto a 320 ml column of Superdex 75 (GE Healthcare Life Sciences), which had been pre-equilibrated with buffer containing: 20mM Hepes pH 7.4, 10% glycerol, 500mM NaCl, for gel filtration. The purified protein was divided into aliquots and stored at -80°C.

### PolyIC electrophoretic mobility shift assay (EMSA)

Low molecular weight (LMW) polyIC (Invitrogen) was labeled with Cy3 using the Label IT® nucleic acid labeling kit (Mirus) according to the manufacturer’s protocol. 0.5 μg of labeled polyIC was incubated for 30 minutes with increasing amounts of purified dsRBEC (0.5–4 μg), followed by electrophoresis of the mixture on a 1.5% TAE-agarose gel. The gel was visualized using the MF-ChemiBIS system (DNR Bio-Imaging Systems).

### ^125^I-EGF displacement assay

A431 cells were harvested by trypsinization and resuspended in PBS supplemented with 1% BSA and plated in 96-well MultiScreen filter plates (Millipore) (5,000 cells per well). Following 30 minutes incubation on ice with gentle shaking, the medium was aspirated using a MultiScreen^HTS^ vacuum manifold (Millipore) and replaced with ice-cold PBS supplemented with 0.1% BSA. Increasing concentrations (0–16 nM) of the dsRBEC or hEGF (PeproTech) were added to the wells, in triplicate. Following 30 minutes incubation on ice, the cells were supplemented with ^125^I-EGF (0.1 nM, PerkinElmer) and incubated 4 h longer on ice, with gentle shaking. The medium was then aspirated, and the cells were washed five times with ice-cold PBS supplemented with 0.1% BSA and the plate was left under vacuum for complete drying. The MultiScreen plate was then exposed to a phosphor imager plate (BAS-IP MS 2040 Fuji Photo Film) for 72 hours. An ^125^I-EGF calibration curve (0–10 fmol) was used to convert pixels into absolute concentrations. The plate was scanned using a FujiFilm Fluorescent Image Analyzer FLA-3000. Nonlinear regression (competitive binding one site analysis) was performed on the data using GraphPad Prism^TM^, Version 5.0. Kd values were calculated as the means from three independent binding experiments, each of which comprised triplicate samples. The Kd of ^125^I-EGF, which was required for the analysis of the displacement data, was measured as previously described by Abourbeh et al [22] (S1 Fig).

### EGFR phosphorylation

MDA-MB-468 cells were plated in 6-well plates (500,000 cells per well) in RPMI 1640 medium and 10% FCS. 24 hours after plating, the cells were washed twice with PBS and the medium was replaced with RPMI medium lacking FCS. 16 hours later, the cells were treated with dsRBEC a various concentrations for 15 minutes. The cells were then harvested with hot Laemmli sample buffer. EGFR phosphorylation was evaluated by western blot analysis, using anti-phospho (Y1068)-EGFR antibody (Cell Signaling Technology, # 2234, 1:1000 dilution), anti-EGFR antibody (Santa Cruz, sc-03, 1:1000 dilution) and anti-GAPDH antibody (Santa Cruz, sc-25778, 1:2000 dilution).

### Confocal microscopy

MDA-MB-468 (10000 cells/well) and MCF7 (7000 cells/well) cells were plated in a μ-Slide 8 Well Glass Bottom plate (Ibidi). 48 hours after plating, the medium was replaced with fresh medium containing 1μM sulforhodamine G (Biotium Inc.) and the plate was placed in the 37℃ chamber of a confocal microscope (FV-1200 Olympus, Japan). The cells were then treated with 1μg/ml Cy3-polyIC alone, or 1μg/ml Cy3-polyIC which had been pre-incubated with dsRBEC (polyIC:dsRBEC, weight:weight ratio of 1:2). PolyIC internalization was monitored for 2 hours. For endosomal localization we treated MDA-MB-468 cells with polyIC/dsRBEC (as described above) and 5μg/ml AlexaFluor 647-labeled transferrin (Jackson ImmunoResearch Laboratories, Inc) simultaneously.

### EGFR Level

The expression of EGFR in MDA-MB-468 and MCF7 cells was analyzed by western blot using anti-EGFR antibody (Santa Cruz, sc-03, 1:1000 dilution). Anti-GAPDH antibody (Santa Cruz, sc-25778, 1:2000 dilution) was used a loading control. Expression of surface EGFR in the two cell lines was assessed by flow cytometry using PE-conjugated anti-human EGFR antibody (BioLegend, # 352903).

### Survival assay

MDA-MB-468 and MCF7 cells were plated in 96-well plates (5000 and 2000 cells per well, respectively). The next day the medium was refreshed and the cells were treated with polyIC, dsRBEC or polyIC which had been pre-incubated with dsRBEC (polyIC:dsRBEC weight:weight ratio of 1:2). 72 hours following the treatment, the survival of the cells was measured by the methylene blue colorimetric assay [23].

### Apoptosis

#### FACS

MDA-MB-468 and MCF7 cells were plated in a 24-well plate (150,000 and 100,000 cells per well, respectively).The following day the cells were treated with polyIC (1μg/ml), dsRBEC (2μg/ml) or polyIC (1μg/ml) which had been pre-incubated with dsRBEC (polyIC:dsRBEC weight:weight ratio of 1:2) for 8 hours. Annexin V/Propidium iodide (PI) staining was performed using the MBL MEBCYTO apoptosis kit according to the manufacturer's guidelines and analyzed using flow cytometry, BD FACS ARIAIII (BD Biosciences, USA).

#### Caspase-3 and PARP cleavage

MDA-MB-468 cells were plated in 12-well plates (250,000 cells per well). The next day the medium was refreshed and the cells were treated with polyIC (1μg/ml), dsRBEC (2μg/ml) or polyIC (1μg/ml) which had been pre-incubated with dsRBEC (polyIC:dsRBEC weight:weight ratio of 1:2). 4 hours following the treatment, the cells were harvested with hot Laemmli sample buffer and subjected to SDS–PAGE (12% polyacrylamide) and western blotting., using anti-Cleaved Caspase-3 antibody (Cell Signaling Technology, #9661, 1:1000 dilution), anti-PARP antibody (Cell Signaling Technology, #9542, 1:1000 dilution) and anti-GAPDH antibody (Santa Cruz, sc-25778, 1:2000 dilution).

### RNA isolation and reverse transcriptase–real time polymerase chain reaction (qRT-PCR)

MDA-MB-468 cells were treated with polyIC (1μg/ml), dsRBEC (2μg/ml) or polyIC (1μg/ml) which had been pre-incubated with dsRBEC (polyIC:dsRBEC weight:weight ratio of 1:2) for 2 and 4 hours. Total RNA was isolated from MDA-MB-468 cell using the EZ-10 DNA away RNA- Mini-prep Kit (Bio Basic) according to the manufacturer's instructions. 1 μg of total RNA was reverse-transcribed using the High-Capacity cDNA Reverse Transcription Kit (Applied Biosystems) and the resulting cDNA was used for qRT-PCR analysis (Fast SYBR Green; Applied Biosystems) using the primer pairs listed in Table 1. Gene expression was normalized to GAPDH gene expression and compared to samples from vehicle-treated cells. Fold change was quantified using the ^ΔΔ^ CT method.

**Table 1:** qRT-PCR primer sequences.

Gene	Primer sequence
GAPDH	F: 5' GAGCCACATCGCTCAGAC 3'
	R: 5' CTTCTCATGGTTCACACCC 3'
IFN-β	F: 5' ATGACCAACAAGTGTCTCCTCC 3'
	R: 5' GCTCATGGAAAGAGCTGTAGTG 3'
CCL5	F: 5' CGCTGTCATCCTCATTGCTACTG 3'
	R: 5' GCAGGGTGTGGTGTCCGAG 3'
IP10	F: 5' GCCAATTTTGTCCACGTGTTG 3’
	R: 5' AGCCTCTGTGTGGTCCATCCT 3'
TNFα	F: 5' GTGCTTGTTCCTCAGCCTCTT 3’
	R: 5' GGCCAGAGGGCTGATTAGAGAG 3’

### ELISA

MDA-MB-468 and MCF7 cells were plated in 96-well plates (10,000 and 7,000 cells per well, respectively). The next day the medium was refreshed and the cells were treated with polyIC, dsRBEC or polyIC which had been pre-incubated with dsRBEC (polyIC:dsRBEC weight:weight ratio of 1:2). 24 hours following the treatment, the medium was collected. Interferon gamma-induced protein 10 (IP10), chemokine (C-C motif) ligand 5 (CCL5) and tumor necrosis factor alpha (TNFα) proteins were quantified using ABTS ELISA Development Kits (PeproTech) according to the manufacturer’s protocol. Interferon beta (IFN-β) protein was quantified using a bioluminescent ELISA kit (LumiKine) according to the manufacturer’s protocol.

### Statistical analysis

GraphPad Prism was used for all statistical analysis. One-way ANOVA and Tukey post test were used to analyze the ELISA experiments. The survival assay was analyzed using two-way ANOVA and Bonferroni post test analysis.

## Results

### Expression and purification of dsRBEC

We designed a chimeric protein vector, dsRBEC, for the targeted delivery of polyIC to EGFR over-expressing tumor cells. This vector comprised the dsRBD of human PKR fused via a linker to human EGF (see Materials and Methods and Fig 1).

**Fig 2:**
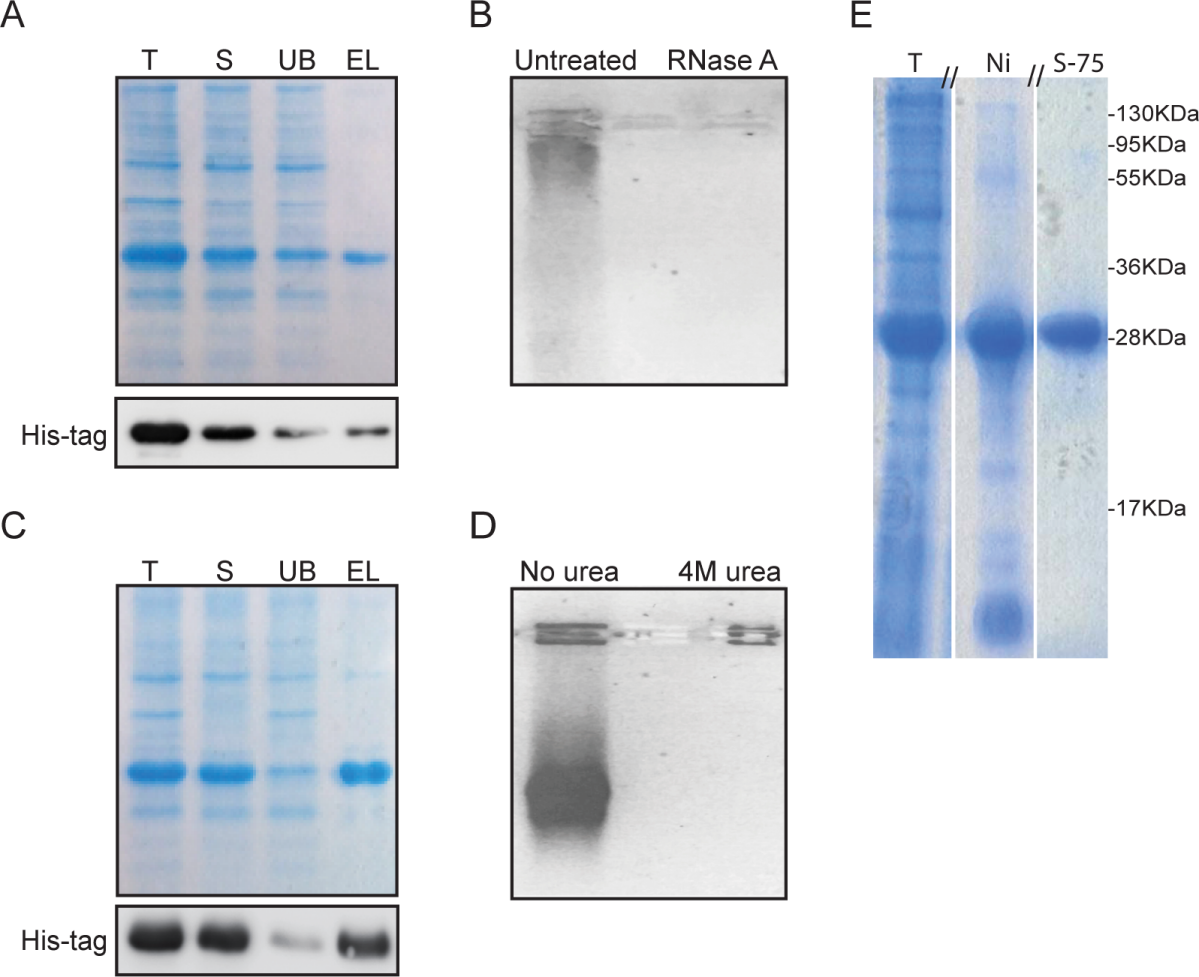
Purification of dsRBEC. **A)** dsRBEC was purified on Ni Sepharose under native conditions. Samples of total lysate (T), soluble fraction (S), unbound fraction (UB) and eluate (EL) were electrophoresed by SDS-PAGE. dsRBEC was visualized with Coomassie dye and by western blot analysis using an anti-His antibody. **B)** Samples of EL electrophoresed on 1% agarose and stained with ethidium bromide. Treatment with 10μg/ml RNase A eliminated staining. **C)** dsRBEC was purified on Ni Sepharose under denaturing conditions ***(***4M urea). Samples of total lysate (T), soluble fraction (S), unbound fraction (UB) and eluate (EL) were analyzed as in (A). **D)** Equal amounts of eluted protein, isolated under native or denaturing conditions, were electrophoresed and stained with ethidium bromide. **E)** SDS-PAGE analysis of final dsRBEC purification, (15% bis-acrylamide gel stained with Coomassie dye) T, total crude lysate before purification; Ni, eluate from 4 ml Ni Sepharose column; S-75, eluate from final purification on Superdex75 (Uncropped gel can be visualized in S2 Fig).

dsRBEC was efficiently expressed as a His_6_-tagged protein in *E*. *coli* BL21(DE3)/CodonPlus RIL. As a first step, the chimera was purified using Ni Sepharose High Capacity resin. Under native conditions the yield of protein was very low, due to poor solubility and poor binding to the resin (Fig 2A). Furthermore, the purified dsRBEC was contaminated with nucleic acids, as indicated by the high OD_260_/OD_280_ ratio of 1.92 and by ethidium bromide staining. When we treated the eluted protein with RNase A prior to electrophoresis, ethidium bromide staining was no longer detectable (Fig 2B). This indicated that the contaminating nucleic acid was host RNA bound to dsRBEC, presumably at its dsRBD.

In order to remove the contaminating RNA under native conditions, we took several approaches, including treatment with RNase A or polyethyleneimine (PEI). However, these treatments resulted in precipitation of our protein. We therefore attempted to purify the chimera under denaturing conditions. We lysed the bacteria and bound the lysates to Ni Sepharose beads in the presence of 4M urea. Under these conditions, the protein was highly soluble and bound the column with increased affinity (Fig 2C). The amount of contaminating RNA in the eluate was significantly reduced, as detected by the decrease in OD_260_/OD_280_ ratio to 0.7 and by the lack of staining with ethidium bromide (Fig 2D). Thus, denaturing conditions facilitated the removal of the contaminating RNA, improved the solubility of the chimera and increased its yield.

The next step was to scale up the purification procedure. To process larger amounts of lysate, we decided to use the AKTA Explorer system. This system made it very easy to perform on-column refolding, with a continuous, gradual reduction in urea concentration, while the protein was still bound to the Ni Sepharose resin. The immobilization of a protein on a column prevents protein aggregation caused by intermolecular interactions [24–27]. To remove remaining protein impurities (Fig 2E Lane 2), the eluate from the Ni Sepharose column was subjected to a second purification step, Superdex75 gel filtration (Fig 2E Lane 3). The total yield of purified protein was 6 mg from 0.5L of bacterial culture.

### dsRBEC binds polyIC

**Fig 3:**
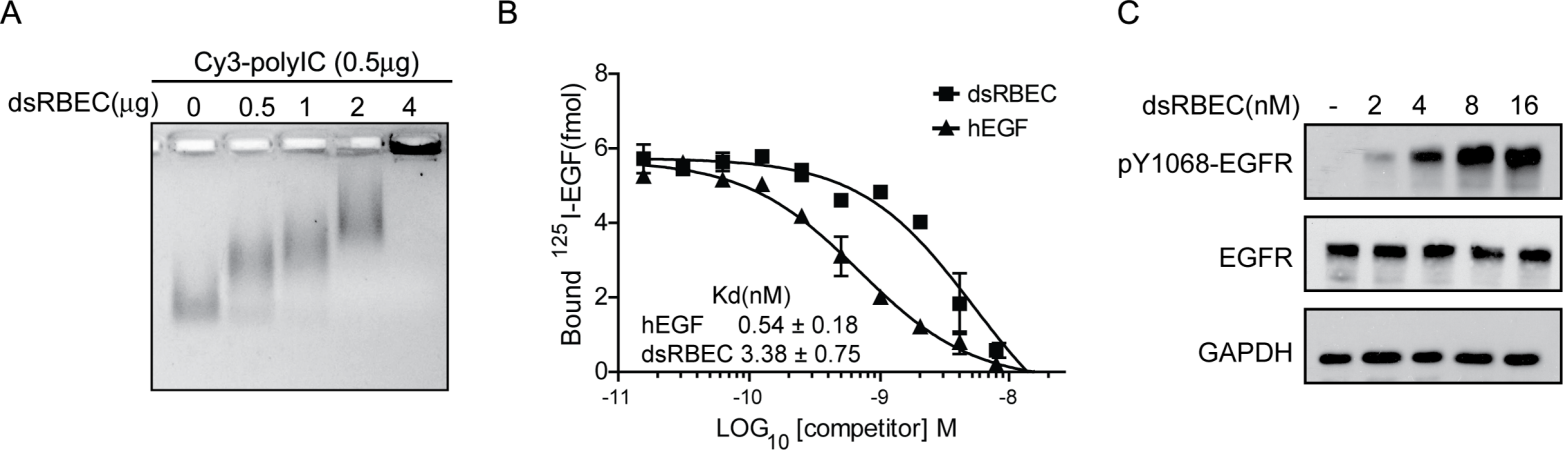
Analysis of dsRBEC activity. **A)** EMSA showing reduced mobility in 2% agarose of polyIC/dsRBEC complexes. Lane 1, polyIC (0.5 μg); Lanes 2–5, polyIC (0.5 μg) pre-incubated with dsRBEC (0.5–4 μg). **B)** Displacement of ^125^-I EGF by dsRBEC (■) or unlabeled hEGF (▲) in A431 cells. The graph shows means ±SD from a representative experiment, performed using duplicate samples. The Kd was calculated as the mean from three independent experiments. **C)** Western blot analysis of EGFR phosphorylation following treatment of MDA-MB-468 cells with dsRBEC at various concentrations for 15 minutes.

To evaluate the ability of dsRBEC to bind polyIC, we performed an EMSA test. Incubation of dsRBEC with Cy3-labeled polyIC (Cy3-polyIC) retarded the migration of Cy3-polyIC on a 2% agarose gel in a dose-dependent manner (Fig 3A), showing that the dsRBD moiety of dsRBEC is competent to bind polyIC.

### dsRBEC binds EGFR with high affinity

The affinity of the purified dsRBEC to EGFR was measured by competitive binding experiments, using radio-labeled EGF. We measured the decrease in ^125^I-EGF binding in the presence of increasing concentrations of unlabeled hEGF or of dsRBEC (Fig 3B). Kd values of 0.54±0.18 nM for hEGF and 3.38±0.75 nM for dsRBEC were calculated using competitive binding one site analysis (GraphPad Prism 5). dsRBEC retains high affinity to EGFR, suggesting that the N-terminal fusion to the dsRBD moiety did not detract from the affinity of the EGF moiety for EGFR, and that the refolding protocol regenerated the active conformation.

EGF binding to EGFR leads to receptor autophosphorylation, followed by clathrin-coated pit mediated endocytosis [28]. To test for productive binding of dsRBEC to EGFR, we measured the autophosphorylation of tyrosine residue 1068 of EGFR, a characteristic EGFR autophosphorylation site, which is critical for EGFR internalization [28]. Since A431 cells have higher levels of basal EGFR phosphorylation [29], we used MDA-MB-468 cells, which also express high levels of EGFR, but show much less basal phosphorylation [30]. dsRBEC treatment for 15 minutes induced the phosphorylation of tyrosine 1068 in a dose-dependent manner (Fig 3C). This indicates that dsRBEC binds EGFR correctly and can induce receptor phosphorylation, which is necessary for internalization.

### dsRBEC selectively induces polyIC internalization in EGFR over-expressing cells

**Fig 4:**
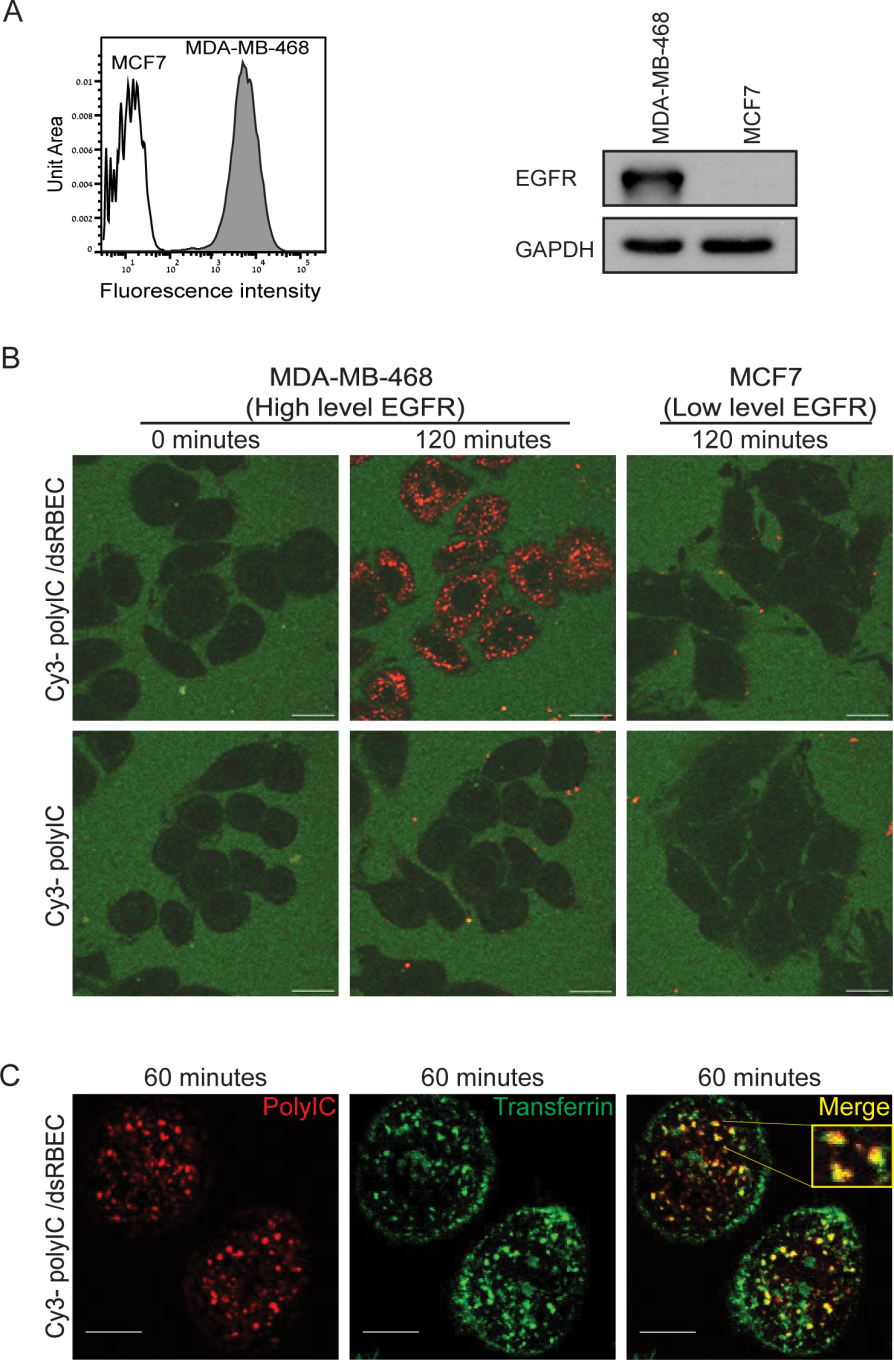
dsRBEC selectively introduces polyIC into EGFR over-expressing cells. **A)** Expression of EGFR in MDA-MB-468 and MCF7 cells was evaluated by FACS (left) or by western blot (right) as described in the Materials and Methods. **B)** Confocal live imaging of Cy3-polyIC internalization in MDA-MB-468 and MCF7 cells. Cy3-polyIC was delivered by dsRBEC (Upper row), or added directly to the cell culture medium (Lower row). The figure shows Cy3-polyIC localization at time 0 (before treatment) and after 120 minutes of treatment (Scale bar 20μm). **C)** Cy3-polyIC/dsRBEC and AlexaFluor647-transferrin were added to MDA-MB-468 cells simultaneously. Endosomal localization of Cy3-polyIC/dsRBEC is indicated by its strong co-localization with the recycling endosomal marker transferrin, 60 minutes after the start of treatment. Cy3-polyIC (red), transferrin (green), merge (yellow). (Scale bar 10μm).

Next, we tested whether dsRBEC could deliver polyIC selectively into EGFR over-expressing cells. We compared MDA-MB-468 cells, which over-express EGFR, and MCF7 cells, which express low or undetectable levels of the receptor [31] (Fig 4A). Using confocal microscopy we demonstrated that Cy3-polyIC complexed with dsRBEC (Cy3-polyIC/dsRBEC) was internalized into MDA-MB-468 cells, whereas no internalization was observed in MCF7 cells (Fig 4B). Naked Cy3-polyIC was not internalized by either cell line (Fig 4B). The punctate fluorescent pattern of Cy3-polyIC when carried by dsRBEC is indicative of endosomal entrapment [32–34]. To verify endosomal localization we treated MDA-MB-468 cells with Cy3-polyIC/dsRBEC in the presence of AlexaFluor647-conjugated transferrin, a recycling endosomal marker. Indeed, we observed strong co-localization of Cy3-polyIC with transferrin (Fig 4C). These data corroborate our findings that the dual functional chimera, dsRBEC, can bind both EGFR and polyIC. Thus, dsRBEC induces targeted, selective delivery of polyIC into EGFR over-expressing cells, where it accumulates in endosomes.

### Targeted delivery of polyIC by dsRBEC leads to apoptosis of MDA-MB-468 cells

**Fig 5:**
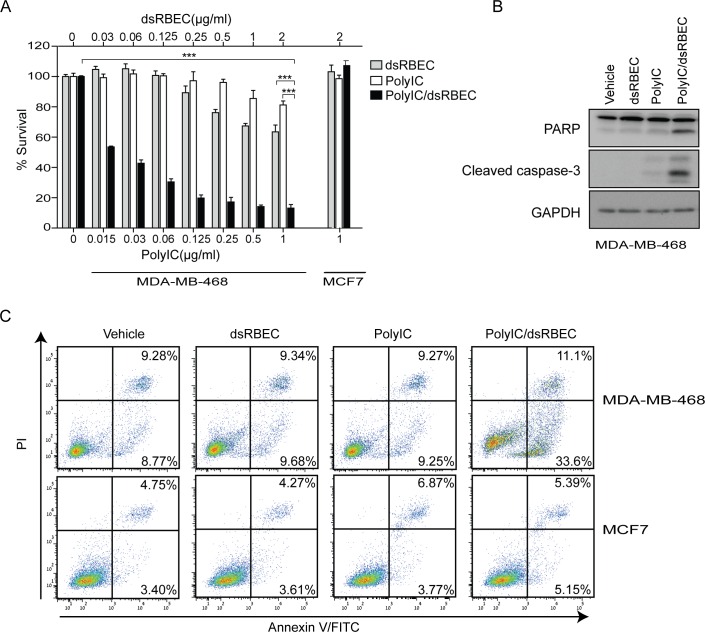
PolyIC/dsRBEC induces apoptosis in MDA-MB-468 but not in MCF7 cells. **A)** Survival following treatment with dsRBEC alone, polyIC alone or polyIC/dsRBEC for 72 hours was analyzed using methylene blue. Viable cells are presented as percent of vehicle-treated control (0). Upper x-axis shows concentration of dsRBEC; lower x-axis shows concentration of polyIC. For MDA-MB-468 cells, the difference between treatment with PolyIC/dsRBEC vs PolyIC alone, or vs dsRBEC alone, or versus vehicle was significant (P<0.0001) for all concentrations tested, but is shown in the figure only for 1μg/ml polyIC, for ease of presentation. **B)** Apoptosis in MDA-MB-468 cells was evaluated by western blot showing cleaved PARP and caspase 3 following treatment with dsRBEC alone, polyIC alone or polyIC/dsRBEC for 4 hours. **C)** MDA-MB-468 and MCF7 cells were treated with dsRBEC alone, polyIC alone or polyIC/dsRBEC for 8 hours. Annexin V–FITC and PI binding were measured by flow cytometry. The percentage of stained cells is written in the right corner of the relevant quadrant.

We next evaluated the survival of MDA-MB-468 cells, following treatment with polyIC/dsRBEC. PolyIC/dsRBEC led to reduced survival of MDA-MB-468 cells, in a dose-dependent manner, whereas MCF7 cells were unaffected by the treatment even at the highest concentration tested (1μg/ml) (Fig 5A). A complex of 0.5 μg/ml polyIC with 1 μg/ml dsRBEC led to a 90% decrease in survival of MDA-MB-468 cells (Fig 5A). The chimera dsRBEC alone had a slight inhibitory effect on MDA-MB-468 cell survival, which is consistent with earlier reports that EGF alone can mediate apoptosis of this cell line [35,36]. Notably, naked polyIC had a much weaker effect on cell survival than polyIC/dsRBEC (***,P<0.0001 for effect of polyIC/dsRBEC vs polyIC alone, for polyIC/dsRBEC vs dsRBEC alone and for polyIC/dsRBEC vs vehicle at all tested concentrations).

We next tested whether the decreased cell survival upon treatment with PolyIC/dsRBEC was due to apoptosis. In MDA-MB-468 cells treated with polyIC/dsRBEC, cleavage of both PARP and caspase-3 was evident as soon as 4 hours after treatment initiation (Fig 5B). Furthermore, there was a strong increase in Annexin V-positive cells after 8 hours of treatment with polyIC/dsRBEC (Fig 5C). In contrast, treatment with naked polyIC or dsRBEC alone had no or little effect on PARP or caspase-3 cleavage (Fig 5B) or on Annexin V staining (Fig 5C). MCF7 cells were not affected by the treatment and did not show any sign of apoptosis (Fig 5C). These results indicate that polyIC/dsRBEC, but not polyIC alone, induces selective apoptosis of EGFR-over-expressing cells.

### PolyIC delivery by dsRBEC induced the expression of pro-inflammatory cytokines from MDA MB-468 cells

**Fig 6:**
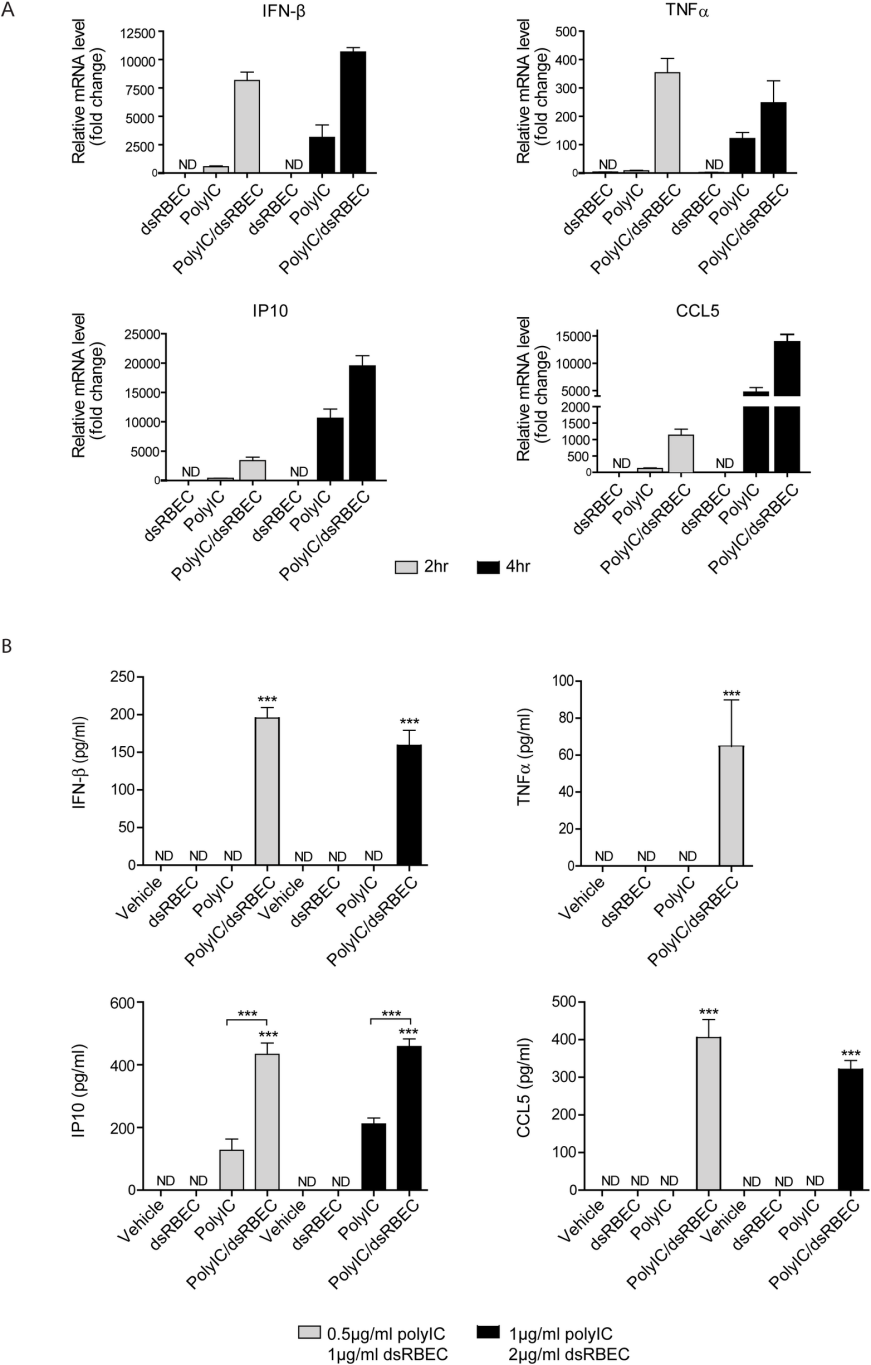
PolyIC/dsRBEC induces the expression and secretion of pro-inflammatory cytokines in MDA-MB-468 cells. **A)** qRT-PCR analysis of IFN-β, CCL5, IP10 and TNFα mRNA expression following treatment with dsRBEC alone, polyIC alone or polyIC/dsRBEC for 2 and 4 hours. Data were normalized to GAPDH and are expressed as fold change relative to vehicle-treated samples. A representative experiment out of 3 experiments is shown. Error bars represent RQ max. **B)** Protein levels of IFN-β, CCL5, IP10 and TNFα were measured by ELISA following treatment with dsRBEC alone, polyIC alone or polyIC/dsRBEC for 24 hours. Values are averages of triplicate biological samples from one representative experiment. (***,P<0.0001 for effect of polyIC/dsRBEC vs polyIC alone, for polyIC/dsRBEC vs dsRBEC alone and for polyIC/dsRBEC vs vehicle).

PolyIC has been reported to stimulate the secretion of pro-inflammatory cytokines [7–9]. We therefore measured the effect of polyIC/dsRBEC treatment on pro-inflammatory cytokine production. MDA-MB-468 cells were treated with polyIC/dsRBEC for 2 or 4 hours, and the mRNAs of IFN-β, IP-10, CCL5 and TNFα were measured using qRT-PCR (Fig 6A). Treatment with polyIC/dsRBEC led to profound induction of cytokine mRNA expression. In contrast, treatment with naked polyIC led to substantially less mRNA expression. Treatment with dsRBEC alone did not affect the expression of these cytokines.

To verify that polyIC/dsRBEC induces increased cytokine secretion, we measured the protein levels of these cytokines using ELISA. After 24 hours of treatment with polyIC/dsRBEC, significant levels of the four cytokines were detected in the medium (Fig 6B). At the highest concentration of polyIC/dsRBEC tested (1μg/ml polyIC, 2μg/ml dsRBEC) TNFα could not be detected, probably because the cells underwent apoptosis before they were able to secrete TNFα. Interestingly, although naked polyIC induced the transcription of all four tested genes (Fig 6A), only IP10 was detected by ELISA, at substantially lower levels than were induced by polyIC/dsRBEC. In addition, none of the tested cytokines were detected in the medium of MDA-MB-468 cells treated with dsRBEC alone (Fig 6B) or in the medium of MCF7 cells in all sets of treatments (Data not shown). Thus, polyIC/dsRBEC induced both the mRNA transcription and protein secretion of pro-inflammatory cytokines in a highly specific manner.

Taken together, our data show that dsRBEC can be efficiently purified from *E*. *coli* in an active conformation. dsRBEC is an efficient vector for targeting polyIC into EGFR over-expressing tumor cells, leading to apoptosis and cytokine secretion.

## Discussion

In the present study we present the development of a novel, recombinant protein carrier, dsRBEC, to selectively deliver polyIC into EGFR over-expressing tumors. dsRBEC is a bifunctional protein, with a dsRBD to bind polyIC and an EGF domain to deliver the polyIC into EGFR over-expressing cells. dsRBEC effectively and selectively induced polyIC internalization into EGFR over-expressing cells, inducing cell death and cytokine secretion. As opposed to current anti-EGFR therapies, which inhibit EGFR activity per se, we exploit EGFR expression and activation as the Achilles’ heel of the tumor. We use the over expression of the receptor as a selective entryway into the cell.

Upon purification from *E*. *coli*, RNA binding proteins are frequently contaminated by non-specific host nucleic acids, thus complicating the protein purification procedure [37,38]. One widely used method to remove nucleic acid contaminants is precipitation with PEI [38–40]. This method, however, is time-consuming and requires the titration of PEI concentration and of the ionic strength [38,41]. In addition, traces of PEI can interfere with the function of the purified protein [40]. Furthermore this method is also difficult to reproduce [38]. RNase and DNase can be used for removal of nucleic acid contamination, but any traces of the nucleases must be removed [37,42]. Applying these methods to our protein resulted in precipitation of dsRBEC.

Marenchino et al. succeeded in purifying His-tagged HIV-1 Rev by destabilizing the protein-RNA interactions using 8M urea, with subsequent on-column refolding [38]. We found that 4M urea was sufficient for the complete removal of contaminating RNA from dsRBEC. Denaturation using low concentrations of urea preserves the native-like protein structure, minimizing the aggregation of protein molecules during refolding, and facilitating recovery of the protein’s biological activity [43]. Furthermore, the lower concentration of urea in our study allowed us to purify the protein at 4°C. This would have been impossible using 8M urea, which crystallizes at cold temperatures. We designed a simple, reproducible on-column refolding procedure, which was easily scaled up using the AKTA Explorer system. This straightforward procedure can be used for other RNA binding proteins. The use of urea to remove RNA contamination also increased dramatically the yield of the purified protein, since it improved the solubility and the binding to nickel resin. Following purification, we confirmed that both domains of the bifunctional chimeric protein were active, by showing that it could bind polyIC efficiently, as well as bind to and activate the EGFR.

In this study, we demonstrate that dsRBEC induces the uptake of polyIC only in cells that over-express the EGFR. Since healthy cells express EGFR at low levels, we expect this treatment to be highly selective, and not to affect non-cancerous cells. Furthermore, naked polyIC has little or no effect on tumor cells at the concentrations that we used. Previous studies have shown the potency of naked polyIC to induce tumor cell apoptosis through the TLR3 pathway. However much higher concentrations (25–50μg/ml) of polyIC were used in these studies [3,4,9]. Although TLR3 is expressed on both the cell surface and the endosomal membrane, it is believed to bind its ligand and undergo proteolytic activation in the endosomal compartment [44–47]. Therefore, we hypothesize that the reason polyIC/dsRBEC is much more potent than naked polyIC, is because polyIC/dsRBEC accumulates in the endosome, where it strongly activates TLR3. Moreover, it has been previously reported that EGFR is essential for TLR3 activation [48], therefore, activation of the receptor by dsRBEC may contribute to the increased potency of polyIC. In addition, we cannot completely exclude the possibility that some of our targeted polyIC reaches the cytoplasm. In the cytoplasm, polyIC can activate other dsRNA-binding proteins, such as PKR, retinoic acid-inducible gene I (RIG-1), and melanoma differentiation-associated gene 5 (MDA5) [49–51], which may contribute to the pro-apoptotic effect caused by the internalized polyIC. Our delivery system could advance the clinical use of polyIC, by allowing the use of lower doses and reducing toxicity.

We demonstrated that polyIC/dsRBEC strongly induces the expression of IFN-β, IP10, CCL5 and TNFα. These cytokines should provide a second line of therapeutic effect, activating and recruiting immune cells to the site of the tumor. Type I IFNs have previously been shown to regulate the activation of innate immune cells, including macrophages, natural killer cells and APCs. Type I IFNs regulate the activation and proliferation of adaptive immune cells, directly via the IFN receptor and indirectly by activation of APCs or by up-regulation of MHC and co-stimulatory molecules [52,53]. Type I IFNs can also directly affect cancer cells, by promoting cell cycle arrest and apoptosis [54,55]. Thus, the cytokines produced in response to tumor-targeted polyIC can induce a “bystander” effect, restoring antitumor immune surveillance and eradicating neighboring tumor cells that do not over-express the receptor. Since most tumors are heterogenic, this bystander effect is extremely beneficial.

In conclusion, we have developed a bi-domain recombinant chimeric protein that is capable both of binding polyIC and of delivering it into EGFR over-expressing tumors, inducing tumor cell death. On the basis of this study, we suggest that dsRBEC-delivered polyIC may be a promising therapy for EGFR over-expressing tumors. We have created a platform technology, with dsRBEC as the prototype recombinant protein. By replacing the EGF moiety with targeting moieties for different receptors, this approach can be applied to additional tumor types.

## Supporting Information

S1 Fig ^125^I-EGF Binding in A431 cells. A431 cells were incubated with 0–2,000 pM ^125^I-EGF for 4 hours at 4 ℃. Total: total binding, NSB: non-specific binding; SB: specific binding. Non-specific binding was measured in the presence of 1μM unlabeled hEGF. The data were analyzed using GraphPad Prism 5, yielding a Kd value of 138.3±30.63 pM.
(TIF)

S2 Fig Uncropped gels showing dsRBEC purification. **A) Ni Sepharose column.** Lane 1, insoluble pellet following bacterial lysis; Lane 2, soluble lysate before purification (= T in Fig 2E); Lane 3, unbound protein; Lane 4, molecular weight markers; Lanes 5–13 and 15 fractions eluted from Ni Sepharose (Lane 8 = Ni in Fig 2E), Lane 14, Pool of fractions represented in 8–11. **B) Superdex75 gel filtration**. Pooled fractions 8 through 11 were loaded onto 320 ml Superdex 75. Lane 1, same as lane 14 in gel A. Lanes 2–15 eluates collected from the column. The eluates in Lanes 7–11 were pooled, divided into aliquots and used for subsequent analysis. (Lane 8 = S-75 in Fig 2E).
(TIF)
