# Supplementary figures and images for: Targeting polyIC to EGFR over-expressing cells using a dsRNA binding protein domain tethered to EGF
Source: PLoS One. 2016 Sep 6;11(9):e0162321. doi: 10.1371/journal.pone.0162321 (PMC5012564; doi:10.1371/journal.pone.0162321)

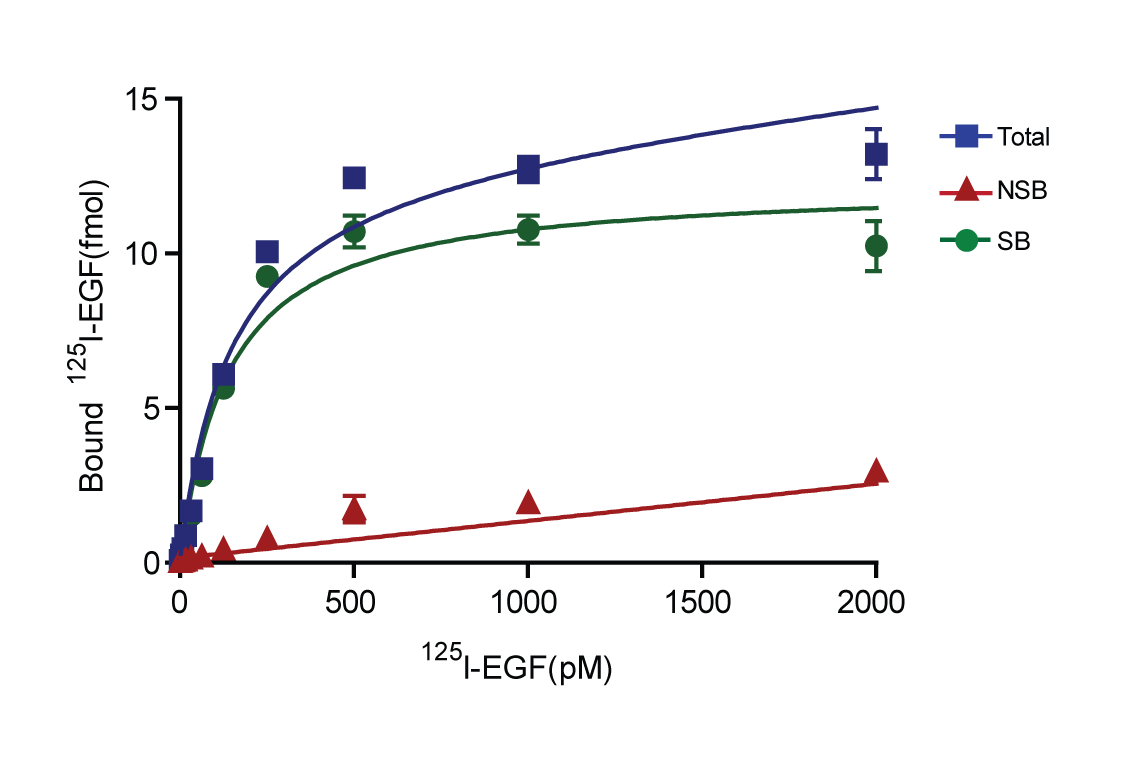

Supplement: S1 Fig — A431 cells were incubated with 0–2,000 pM 125I-EGF for 4 hours at 4 ℃. Total: total binding, NSB: non-specific binding; SB: specific binding. Non-specific binding was measured in the presence of 1μM unlabeled hEGF. The data were analyzed using GraphPad Prism 5, yielding a Kd value of 138.3±30.63 pM. (TIF) [file pone.0162321.s001.tif]

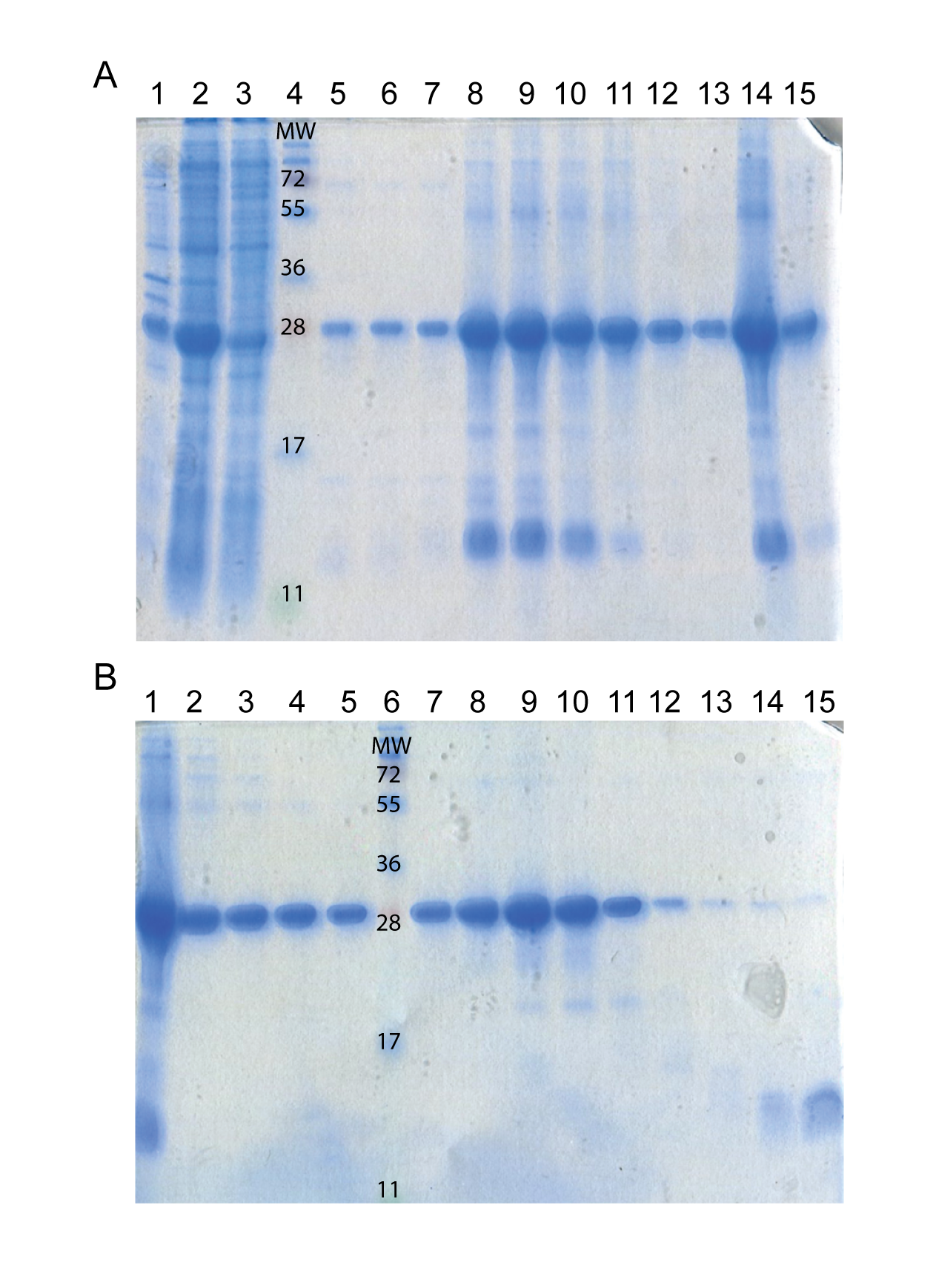

Supplement: S2 Fig — A) Ni Sepharose column. Lane 1, insoluble pellet following bacterial lysis; Lane 2, soluble lysate before purification (= T in Fig 2E); Lane 3, unbound protein; Lane 4, molecular weight markers; Lanes 5–13 and 15 fractions eluted from Ni Sepharose (Lane 8 = Ni in Fig 2E), Lane 14, Pool of fractions represented in 8–11. B) Superdex75 gel filtration. Pooled fractions 8 through 11 were loaded onto 320 ml Superdex 75. Lane 1, same as lane 14 in gel A. Lanes 2–15 eluates collected from the column. The eluates in Lanes 7–11 were pooled, divided into aliquots and used for subsequent analysis. (Lane 8 = S-75 in Fig 2E). (TIF) [file pone.0162321.s002.tif]
